# Does *Crocus sativus* (Saffron) Have an Influence or Effect on Ocular Health: A Systematic Literature Review

**DOI:** 10.1177/2515690X261478846

**Published:** 2026-08-13

**Authors:** Sonia Ferrara, Janet Schloss

**Affiliations:** 1 National Centre for Naturopathic Medicine (NCNM), Faculty of Health, 4571 Southern Cross University, Lismore, NSW, Australia

**Keywords:** ocular health, eye health, ocular, eye, *Crocus sativus*, saffron

## Abstract

**Background:**

*Crocus sativus* (Saffron) and its bioactive constituents, crocin and crocetin have gained attention for their neuroprotective and antioxidant properties in ocular health. This review synthesises clinical evidence from extracted studies evaluating their efficacy, safety and dosing across Age-related Macular Degeneration (AMD), Primary Open-Angle Glaucoma (POAG), Diabetic Macular Edema (DME), Central Serous Retinopathy (CSR), Stargardt Disease (STGD1) and Paediatric Myopia (PM).

**Methodology:**

A systematic literature review was undertaken assessing randomized trials, observational or cohort studies, case studies or series written in English between January 2015 to January 2025. Studies were identified using PubMed, AMED, CINHAL, ScienceDirect and Scopus as well as the grey literature.

**Results:**

Ten clinical trials were identified from 529 citations. Interventions involved oral supplementation of saffron (20-50mg/day), crocin (5-15mg/day) and crocetin (7.5mg/day) over durations ranging from 6 weeks to 12 months. Primary outcomes included visual acuity, retinal electrophysiology, intra-ocular pressure, macular thickness, and safety profiles.

**Conclusion:**

Saffron, crocin and crocetin, demonstrate consistent efficacy and exceptional safety profiles across a range of ocular conditions. Saffron’s dose-dependent therapeutic effects are a promising adjunctive therapy in ophthalmology. Further large-scale trials are warranted to confirm these findings and refine condition specific dosing.

## Introduction

*Crocus sativus*, commonly known as saffron, is a flowering plant renowned for its culinary and medicinal properties. Rich in bioactive compounds such as crocin, crocetin and safranal, saffron exhibits antioxidant, anti-inflammatory, and neuroprotective effects particularly in ocular health.^1^ Additionally, it is high in antioxidants and is believed to protect retinal cells from oxidative stress and inflammation, which are key contributors to ocular health.^1,2^ Emerging evidence suggests potential benefits in retinal protection, improving visual acuity, and slowing the progression of degenerative eye diseases.^3^ However, these findings remain scattered across various small studies and lack systematic synthesis. The exploration of Saffron as a natural intervention for ocular health represents a promising approach in combating vision related challenges which underscores the importance of continued research.

With rising prevalence of age-related eye conditions such as macular degeneration, glaucoma, diabetic retinopathy, including visual impairments affecting individuals across all age groups, the importance of preserving visual function has never been greater. These conditions can significantly reduce quality of life and increase socioeconomic burdens through healthcare costs.^4^ Although current approaches to ocular health offer limited standalone interventions, saffron is often included as part of broader supplement formulations rather than being rigorously evaluated on its own. However, recent clinical trials such as Mahdiani et al.^3^ are beginning to isolate and assess the effects of crocin, saffron’s active compound, in targeted eye conditions such as primary open-angle glaucoma and prompting the need to explore safe and natural therapeutic alternatives for Saffron.

Whilst, globally the burden of vision impairment affects people of all ages, with at least 2.2 billion people affected and 1 billion cases potentially preventable.^5^ There is currently no integrated framework that consolidates existing knowledge about saffron’s effects on ocular health. The research landscape is fragmented, with inconsistent methodologies and variable outcome measures. Additionally, specific research gaps such as optimal dosages, long-term efficacy, and mechanisms of action have not been clearly defined.

A narrative review conducted in 2019 by Heitmar et al.^6^ discusses the mechanistic insights and therapeutic potential of saffron for ocular health. It includes both preclinical (animal and in vitro) and clinical (human) research on saffron’s effects in ocular health disorders.^6^ This review gives background to our review, focusing specifically on the mechanisms, dosage, delivery methods and clinical outcomes across a broader spectrum of ocular conditions, including Age related macular degeneration (AMD), primary open angled glaucoma (POAG), central serous chorioretinopathy (CSR), dry-type age related macular degeneration (AMD), pediatric myopia, Stargardt macular dystrophy (STGD1), refractory diabetic macular edema (DME) and Paediatric Myopia. This distinction fills a critical gap in consolidating evidence for saffron’s ophthalmic applications as it concentrates exclusively on human clinical trials which translates to patient care.

Our review aims to guide future research and clinical applications in this field by systematically evaluating saffron’s role in ocular health. The information may provide practitioners and researchers with actionable insights into therapeutic potential. This review not only bridges complementary medicine and modern ophthalmology but may also open the door to safer, natural adjuncts in eye care.

## Methodology

### Study Aims

Our study is guided by three core aims:

1. To assess the therapeutic effects of saffron in promoting and preserving ocular health.
2. To identify the specific eye conditions that have been investigated in relation to saffron such as age-related macular degeneration (AMD), diabetic macular edema (DME), and primary open angled glaucoma.
3. To evaluate the efficacy and safety of saffron-based interventions across these conditions, providing a clearer understanding of its clinical relevance.

### Review Registration

The literature review was registered with PROSPERO^7^ before commencement and the registration ID is **CRD420251046137**. The review follows the PRISMA^8^ reporting guidelines.

### Inclusion and Exclusion Criteria

The SLR included randomized control trials, observational studies, case reports published in the past 10 years and will explore saffron and its influence or effect on ocular health. Most of the relevant research was identified by entering “saffron” into PubMed to determine where the majority of studies were concentrated. In addition, non-English, animal and non-human publications will be excluded from this review.

### PICO Framework

We began by structuring our research question using the PICO model Population, Intervention, Comparison, and Outcome. This helped us define our scope clearly:

• Population: Ocular health in general population
• Intervention(s) or exposure(s): Saffron, Ophthalmological investigations
• Comparator(s) or control(s): Placebo or control or no comparator
• Outcome: Improvements in visual function, retinal health, and safety profiles

This framework guided our inclusion criteria and search strategy, ensuring relevance and precision in study selection.

### Databases

Studies were identified using PubMed, AMED, CINHAL, ScienceDirect and Scopus.

### Search Terms

The following is an example of PubMed search strings and the terms customised for each electronic database. The protocol for the search utilises Boolean operators such as “AND”, “OR”, and combines the following key words: ocular health OR eye health OR eye OR ocular AND saffron OR crocus sativus AND trial OR study OR case report OR observational study OR cohort trial OR cohort study OR clinical trial.

### Data Management

All citations were imported into EndNote^9^ then exported to Covidence for screening. Duplicates were automatically removed when imported into Covidence.^10^ Screening for title and abstract then occurred by authors. From identified citations, full text review was conducted by the authors.

### Risk of Bias

The Joanna Briggs Institute (JBI) checklist was utilised to assess risk of bias for Randomized Control Trials (RCT) and Cohort studies. The risk of bias for RCT, comprises 13 questions (Table 1) and the cohort critical appraisal with 11 questions (Table 2). Only studies scoring 7 or above on the JBI checklist were retained to ensure robust and credible evidence.^11^ The studies were based on their quality. RCT’s produced the most reliable and lease biased conclusions although quality was dependent on the rigour of each research. The controlled studies utilising randomisation to reduce the bias and establish causation despite being, time consuming and ethically or practically limited. The cohort and controlled studies offered valuable observational insight with greater vulnerability to confounding especially in the retrospective designs. Case reports provide detailed descriptions of conditions and treatments and helped generate a hypothesis although their lack of controls limits broader applicability.^12^

**Table 1.** JBI RTC Critical Appraisal Tool & Table

Author and year	Q1	Q2	Q3	Q4	Q5	Q6	Q7	Q8	Q9	Q10	Q11	Q12	Q13	Total
Baldi et al, 2024	Y	N	Y	N	N	N	N	Y	Y	N	N	Y	Y	6
Broadhead et al, 2019	Y	Y	Y	Y	N	Y	Y	Y	Y	Y	Y	Y	Y	12
Hecht et al, 2019	Y	N	Y	Y	N	N	Y	U/S	Y	N	Y	Y	Y	8
Johari et al, 2024	Y	Y	Y	Y	Y	Y	Y	Y	Y	Y	Y	Y	Y	13
Mahdiani et al, 2024	Y	Y	Y	Y	Y	Y	Y	Y	Y	Y	Y	Y	Y	13
Mori et al, 2019	Y	Y	Y	Y	Y	Y	Y	Y	Y	Y	Y	Y	Y	13
Piccardi et al, 2019	Y	Y	Y	Y	Y	Y	Y	Y	Y	Y	Y	Y	Y	13
Riazi et al, 2017	Y	N	Y	N	N	Y	N	Y	Y	Y	N	Y	Y	8
Sepahi et al, 2018	Y	Y	Y	Y	Y	Y	Y	Y	Y	Y	Y	Y	Y	13

Legend: Yes (Y)/No (N)/Unsure (U/S)/Not Applicable (N/A) < 7 excluded.

1. Was true randomization used for assignment of participants to treatment groups?

2. Was allocation to treatment groups concealed?

3. Were treatment groups similar at the baseline?

4. Were participants blind to treatment assignment?

5. Were those delivering the treatment blind to treatment assignment?

6. Were treatment groups treated identically other than the intervention of interest?

7. Were outcome assessors blind to treatment assignment?

8. Were outcomes measured in the same way for treatment groups?

9. Were outcomes measured in a reliable way.

10. Was follow up complete and if not, were differences between groups in terms of their follow up adequately described and analysed?

11. Were participants analysed in the groups to which they were randomized?

12. Was appropriate statistical analysis used?

13. Was the trial design appropriate and any deviations from the standard RCT design (individual randomization, parallel groups) accounted for in the conduct and analysis of the trial?

**Table 2.** JBI Cohort Critical Appraisal Tool & Table

Author and year	Q1	Q2	Q3	Q4	Q5	Q6	Q7	Q8	Q9	Q10	Q11	Total
Broadhead et al, 2024	Y	Y	Y	Y	Y	Y	Y	Y	Y	Y	Y	11
Majeed et al,. 2021	Y	N	Y	Y	Y	Y	Y	Y	Y	Y	Y	10

Legend: Yes (Y)/No (N)/Unsure (U/S)/Not Applicable (N/A)) < 7 excluded.

1. Were the two groups similar and recruited from the same population?

2. Were the exposures measured similarly to assign people to both exposed and unexposed groups?

3. Was the exposure measured in a valid and reliable way?

4. Were confounding factors identified?

5. Were strategies to deal with confounding factors stated?

6. Were the groups/participants free of the outcome at the start of the study (or at the moment of exposure)?

7. Were the outcomes measured in a valid and reliable way?

8. Was the follow up time reported and sufficient to be long enough for outcomes to occur?

9. Was follow up complete, and if not, were the reasons to loss to follow up described and explored?

10. Were strategies to address incomplete follow up utilised?

11. Was appropriate statistical analysis used?

## Results

**Figure 1.**
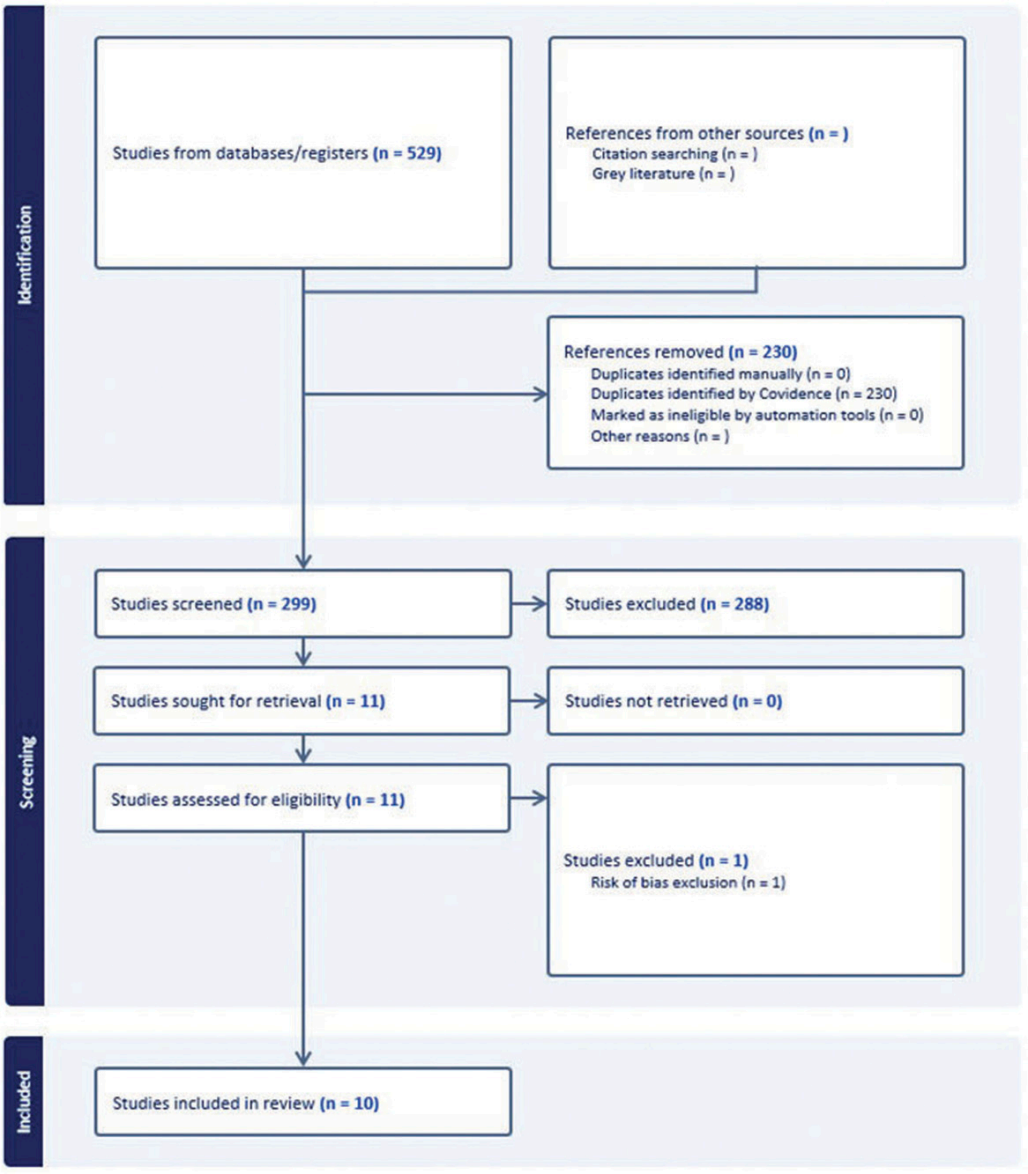
PRISMA Risk of Bias workflow

A systematic review was conducted following PRISMA^8^ guidelines and a total of 529 citations were identified. In addition, 230 duplicate records were removed resulting 299 citations. Title and abstract screening excluded 288 studies, resulting in 11 studies for full text retrieval and eligibility assessment. Of the 11 studies assessed, 1 study was excluded due to high risk of bias, resulting 10 studies being included in the final synthesis. A PRISMA flow diagram summarising the study selection process is presented (Figure 1).

### Critical Appraisal Results

Baldi et al.^13^ was excluded due to failing key criteria on the JBI RCT appraisal which reflected serious concerns across multiple domains of bias. The study failed to meet key methodological criteria, with “No” responses recorded for questions 2, 4, 5, 6, 7, 10, and 11 in Table 1, which determined whether treatment allocation was concealed, if participants, treatment providers and outcome assessors were blinded or if both groups received identical care aside from the intervention, whether follow-up was sufficiently complete with any group differences appropriately analysed and finally if all participants were assessed within the groups to which they were originally randomized. Hence the study did not meet the minimum quality criteria and was excluded to preserve the integrity and reliability of the evidence base.

All other 10 studies from the JBI appraisals, achieved a score greater than 7 on the JBI checklist and were therefore included in the final synthesis ( Tables 1 and 2).

### Overview Results

The data from the 10 studies have been extracted into a data extraction table (see Table 3). The information from these studies have been discussed under each different eye condition. Interventions involved oral supplementation of saffron (20-50mg/day), crocin (5-15mg/day), and crocetin (7.5mg/day) over durations ranging from 6 weeks to 12 months. Primary outcomes included visual acuity, retinal electrophysiology, intraocular pressure, macular thickness and safety profiles.

**Table 3.** Data Extraction Table

Author & Year (Country)	What methodology did the study use?	What population did study use? (Patient demographics - age, sex, ethnicity, diseases/conditions)	Intervention (quantity, dosage, route of administration, format, duration, time frame, setting)	What the study is Assessing	What is/are outcome(s) of the study?
Broadhead et al, 2019 (Australia)	Randomized, double-blind, placebo-controlled crossover trial	n=100 adults over 50 years with mild to moderate age-related macular degeneration (AMD) and visual acuity better than 20/70 in at least one eye.	Participants received 20 mg/day of saffron or placebo for 3 months, followed by a crossover to the other treatment for another 3 months.	To evaluate the efficacy and safety of oral saffron supplementation in treating mild to moderate age-related macular degeneration (AMD).	Saffron supplementation modestly improved visual function and retinal response in patients with mild/moderate AMD. Benefits were observed even among those already taking AREDS supplements. Longer-term use may offer greater advantages due to the chronic nature of AMD. Saffron was well tolerated, making it a promising adjunct therapy.
			Participants already using AREDS (Age-Related Eye Disease Study) supplements continued their regimen.		
					• Visual Acuity: Mean BCVA improved on saffron vs placebo • In AREDS users, BCVA improved • mfERG: Latency reduced with saffron. Response density improved by 2.8% in AREDS users • Saffron improved BCVA (+0.69 letters, *p*=0.001) and mfERG latency (-0.17 ms, *p*=0.04); no serious adverse events Safety: No significant difference in adverse events between saffron and placebo groups. No serious saffron-related side effects reported.
Broadhead et al, 2024 (Australia)	open-label extension trial (Cohort)	n=93 adults over 50 years with AMD and vision better than 20/70	Saffron supplementation (20mg/day). Multifocal electroretinogram (mfERG) response density and latency. Best-corrected visual acuity (BCVA). Microperimetry and safety outcomes	12-month efficacy and safety of daily oral saffron supplementation (20 mg/day) in patients with mild/moderate AMD, including those already taking AREDS supplements	Saffron supplementation (20mg/day) showed modest improvements in multifocal electroretinogram (mfERG). mfERG response density improved (*p*<0.001); BCVA worsened slightly (-1.6 letters, *p*<0.05), possibly due to cataract. No serious or adverse reactions were reported. Saffron supplementation preserved retinal function over 12 months, especially in central retinal areas, with minimal side effects. While visual acuity didn’t improve, the findings support saffron’s potential as a safe adjunct therapy for early-stage AMD.
Hecht et al, 2019 (Israel)	Single-blind, parallel-arm Randomized controlled pilot trial	n=22 primary open angled glaucoma (POAG) mean age 69, randomized 1:1	Intervention group: Received guidance on lifestyle changes including: Saffron spice supplementation -to eat a small portion of Saffron spice twice a week so that the package (1 gram) will last the entire month. • High-fiber diet • Caffeine avoidance • Aerobic exercise • Head elevation during sleep Control group: Continued standard therapy Duration: Short-term follow-up	Whether dietary and lifestyle changes can improve intraocular pressure (IOP) and illness perception in patients with primary open-angle glaucoma (POAG). IOP measurements and illness perception via Brief IPQ questionnaire.	Potential intra-ocular pressure (IOP) reductions following dietary and lifestyle changes for POAG.
					Saffron group showed significant IOP reduction (*p*<0.05); no adverse events; pilot data suggest neuroprotective potential.
					While IOP was not significantly affected, the lifestyle interventions positively influenced patients’ perception of their illness and overall well-being. These findings suggest that holistic care may enhance quality of life, even if it doesn’t immediately impact clinical measures like IOP.
Johari et al, 2024 (Iran)	Pilot randomized, double blinded clinical trial	N=40 adults (18-60 years) diagnosed with central serous chorioretinopathy (CSR)	Crocin group: 15mg/day for 8 weeks	Safety and efficacy of crocin in improving anatomical changes in CSR	Crocin compound form of saffron has shown protective effects against inflammation, oxidative stress and damage to retinal pigment epithelium. Potential for a non-invasive treatment.
			Placebo group: 15mg/day for 8 weeks		
					The crocin group showed significant improvements in BCVA and reductions in retinal fluid and thickness compared to placebo. Primary and secondary outcomes included best-corrected visual acuity (BCVA), foveal thickness, subretinal fluid height, and subfoveal choroidal thickness, assessed via OCT imaging
Mahdiani et al, 2024 (Iran)	Randomized, triple-blind, placebo-controlled clinical trial	N=50 primary open angle glaucoma (POAG) 30–71 years with intraocular pressure (IOP)	Groups: Crocin group: 15 mg/day for 4 months	Evaluate the neuroprotective and antioxidant effects of crocin (a saffron-derived compound) in managing POAG and preventing disease progression. Intraocular pressure (IOP) Retinal nerve fiber layer (RNFL) via OCT Cup-to-disc ratio (CDR) Best-corrected visual acuity (BCVA).	Potential neuroprotective and antioxidant effects, may help preserve retinal nerve fibre and IOP reduction and may be useful for prevention of POAG.
			Placebo group: Identical placebo tablets		
					Crocin supplementation may offer a safe and effective adjunct therapy for POAG by lowering IOP and stabilizing optic nerve health. Its antioxidant properties could help slow disease progression, even if visual acuity remains unchanged.
			Follow-up: 4-month treatment + 2-month washout		
					IOP: Significantly reduced in the crocin group at 4 months and 6 months. The IOP value decreased significantly in the crocin group after four (P = 0.0001) and six months (P = 0.003). The difference in CDR changes after four and six months was significant between the two groups (P = 0.00009). The crocin and placebo groups did not have a statistically significant difference regarding the changes in BCVA values and RNFL thickness (P = 0.092) CDR: Showed significant improvement compared to placebo.
					RNFL & BCVA: No significant changes, but RNFL remained stable.
					Safety: No serious adverse effects reported
Majeed et al,. 2021 (India & Bangalore)	Open label clinical Pilot study (Cohort)	N=40 individuals aged 50 years or older with early-stage dry-type age related macular degeneration (dAMD).	Intervention: One capsule of Macumax twice daily for 90 days Supplement composition: ZeaLutein (lutein, zeaxanthin, piperine), Bilberry extract, Saffron extract, Zinc monomethionine. The formulation details such as dosage per ingredient are not disclosed in the published paper	Evaluate the safety and efficacy of Macumax, a phyto-mineral supplement, in patients with early-stage dry-type AMD over a 90-day period	Macumax supplementation, a phyto-mineral nutritional may slow disease progression in early-stage dry AMD and no adverse events reported and improvement in vision.
					Subjective improvements: Significant reduction in diminished and distorted vision from day 60 onward (p < 0.05).
					Objective improvements: Amsler’s grid aberration scores improved: only 40% had abnormalities at day 90 vs. 77.5% at baseline (p < 0.05).
					Safety: No adverse events reported. Macumax supplementation was safe and well-tolerated, with evidence of visual symptom improvement and stabilization of disease progression in early-stage dry AMD patients.
Mori et al, 2019 (Japan)	Multicentre, randomized, double blind, placebo controlled clinical trial	n=69 children (6-12years) with myopia (paediatric)	Crocetin group:7.5mg crocetin daily Placebo group: 7.5mg without crocetin 24 weeks	Whether dietary crocetin can supress myopia progression in children. Crocetin may activate EGR-1 a gene linked to myopia suppression and has antioxidant properties that protect ocular tissues.	Crocetin supplementation showed statistically significant suppression of myopia progression in children.
					Primary outcome Axial length (AL) elongation: Crocetin group: 0.18 ± 0.02 mm increase, Placebo group: 0.21 ± 0.02 mm increase. Statistically significant difference (*p* = 0.046)
Piccardi et al, 2019 (Italy)	Randomized, double-blind, placebo-controlled cross over trial study	N=31 ABCA4-related Stargardt macular dystrophy (STGD1) and visual acuity >0.25	6 months of saffron or placebo, followed by crossover. Central 18° focal electroretinogram (fERG) amplitude and phase Visual acuity	To assess whether oral saffron supplementation (20 mg/day) can preserve central retinal function in patients with ABCA4-related Stargardt disease/fundus flavimaculatus, which involves retinal oxidative damage.	Saffron 20mg supplementation may help central retinal function although visual acuity remained the same and may have potential neuroprotective effects.
					fERG Amplitude & S/N: Saffron group: fERG amplitude remained stable
					Placebo group: significant decline in amplitude and S/N (*p*<0.05)
					Visual acuity and fERG phase: Unchanged across both groups.
					Safety: Saffron was well tolerated with no adverse effects.
					Short-term saffron supplementation was safe and helped preserve central retinal function, suggesting potential neuroprotective effects. Further long-term trials are recommended to explore its role in slowing disease progression.
Riazi et al, 2017 (Iran)	Randomized controlled trial	N=54 dry AMD (23 males, 31 females) with dry AMD (dAMD)	Treatment group: 29 participants received saffron (50 mg/day) Control group: 25 participants received no supplementation	evaluate the effects of daily saffron supplementation (sick/day) over 3 months on visual function in patients with dry AMD Visual acuity Contrast sensitivity Retinal thickness Quality of life via the Melbourne Low Vision Index	Improvement in visual acuity and contrast sensitivity may slow progression and enhance visual function although macular thickness changes were not different. • Visual acuity and contrast sensitivity significantly improved in the saffron group • Retinal thickness: No significant changes between groups • Quality of life: Improved in the saffron group Short-term saffron supplementation may enhance visual function especially contrast sensitivity and slow disease progression in patients with dry AMD. It was also associated with better quality of life and was well tolerated.
Sepahi et al, 2018 (Iran)	Placebo-controlled randomized clinical trial Double-masked, placebo-controlled, phase 2 trial	N=60 patients with refractory diabetic maculopathy. 5 mg or 15 mg crocin tablets per day. 101 eyes from 60 patients with refractory diabetic macular edema (DME) unresponsive to standard treatments (e.g. anti-VEGF injections, steroids, laser therapy).	Crocin 5 mg/day Crocin 15 mg/day Placebo Duration: 3 months	To assess the therapeutic effects of crocin, a saffron-derived antioxidant, on refractory DME a major cause of vision loss in diabetic patients. Best-corrected visual acuity (BCVA) Central macular thickness (CMT) via OCT HbA1c and fasting blood sugar (FBS).	Crocin as a potent antioxidant and neuroprotective for treatment of refractory DME in the short term may be promising alongside standard treatments. The best-corrected visual acuity (BCVA) and central macular thickness (CMT) significant improvements suggesting potential neuroprotective and anti-inflammatory effects.
					Crocin 15 mg/day group showed: significant improvement in BCVA compared to placebo (p<0.05) CMT reduced significantly in the crocin 15 mg group (*p*<0.05), HbA1c decreased in the crocin 15 mg group (*p*=0.024; 95% CI: 0.3–0.96)
					Crocin 5 mg/day group showed clinical improvements, but not statistically significant compared to placebo.
					Safety: No serious adverse effects reported.
					Crocin, especially at 15 mg/day, may offer antioxidant and neuroprotective benefits for patients with refractory DME. It improved visual outcomes and metabolic markers, suggesting potential as a safe adjunct therapy though larger, longer-term studies are needed to confirm its clinical significance.

### Age Related Macular Degeneration (AMD)

The strongest evidence for saffron’s efficacy comes from Broadhead et al.^14,15^ mild to moderate AMD studies. Broadhead et al.^14,15^ demonstrated consistent improvements in retinal function and modest gains in visual acuity, improving by +0.69 letters (*p*=0.001) and multifocal electroretinogram (mfERG) latency, reduced by 0.17ms (*p*=0.04). Furthermore, no serious adverse events reported and long term saffron appears to be safe and beneficial for retinal function and supports saffron’s sustained neuroprotective role, though visual acuity may be confounded by age related lens changes.

Majeed et al.^16^ supports the potential of multi-nutrient formulations, though its open label design and small sample size limit generalisability. The study included 40 individuals aged over 50 years with early-stage dry AMD and a Macumax capsule supplement (lutein, zeaxanthin, saffron, bilberry, zinc) twice daily for 90 days. Subjective vison scores improved significantly (*p*<0.05) and Amsler grid abnormalities reduced from 77.5% to 40%. This pilot study suggests that a multi-nutrient supplement may improve subjective symptoms and retinal stability in early AMD. The results showed significant improvement in vision related symptoms by day 60 and day 90. With no reported adverse effects.

Riazi et al.^17^ RCT included 54 patients with dry AMD (23 males, 31 females) whereby 29 participants with dry AMD received 50mg of oral tablet supplementation of saffron daily for 3 months and the control group included 25 participants receiving no supplement. There was presumed focus on best corrected visual acuity (BCVA) and retinal function and with no significant side effects reported during the 3 month intervention period. Results showed significant improvements in visual acuity and contrast sensitivity in the saffron group, while changes in retinal thickness were not statistically significant.

### Primary Open-Angle Glaucoma (POAG)

Hecht et al.^18^ trial involved patients with POAG who were randomly assigned in a 1:1 ratio to either an intervention or a control group. Nineteen completed the trial and were included in the final analysis with a mean age of 69 years and 63% being female. The intervention group received guidance on specific dietary and modifications in addition to their usual medical treatment while the control group continued with standard therapy alone.

The recommended changes included a higher-fibre diet, limiting caffeine intake, regular aerobic exercise, and elevating the head during sleep, saffron supplementation which was utilized as a food as medicine approach in its natural spice form rather than as a capsule or tablet. The study observed potential IOP reductions following dietary and lifestyle changes for POAG which may improve illness perception and patient wellbeing with fewer symptoms and a greater sense of control compared to the control group. Although IOP decreased slightly in both groups, the difference was not statistically significant (*p*=0.866). Overall while dietary and lifestyle modifications may enhance patients’ psychological wellbeing and perception of their condition they do not appear to have a short-term impact on IOP in individuals with POAG.

Mahdiani et al.^3^ RCT trial included 50 POAG patients (40 completed) aged 30-71 years with elevated IOP. They were enrolled and randomly assigned to receive either a daily 15 mg crocin tablet or a placebo for four months alongside their standard treatment. In this study, IOP showed significant reduction in the group receiving crocin after both four and six months of treatment (*p=*0.0001 and *p=*0.003 respectively). Changes in the CDR between the crocin and the placebo groups were also significantly different over the same period, however no statistically significant differences were observed between the two groups in terms of BCVA and RNFL thickness, though optical coherence tomography indicated that RNFL remained stable throughout. These findings suggest that crocin may serve as a beneficial supplement to help slow the progression of POAG. Furthermore, saffron supplementation may effectively reduce IOP without structural changes over the short term, supporting safety and efficacy.

### Central Serous Chorioretinopathy (CSR)

Johari et al.^19^ investigated 40 adults aged 18-60 years with clinically diagnosed CSR were assigned to receive either crocin (15mg/day) or placebo for 8 weeks. Primary and secondary outcomes included BCVA, foveal thickness, sub retinal fluid height, and subfoveal choroidal thickness, assessed via OCT imaging. The placebo group showed no significant anatomical improvement reported, in comparison the crocin group showed significant improvements in BCVA and reductions in retinal fluid and thickness displaying anatomical retinal changes, suggesting a therapeutic effect on CSR. This pilot study provides promising evidence that crocin supplementation may serve as a safe, non-invasive and biologically active treatment for acute CSR.

### Paediatric Myopia (PM)

Mori et al.^20^ double-blind RCT trial, involved 69 children aged 6- 12 years diagnosed with myopia. Participants received a daily oral capsule containing 7.5mg of crocetin or a placebo over a 24-week period. Children in the crocetin group experienced a smaller increase in axial length compared to those in the placebo group with a difference reaching statistical significance (*p=*0.046). Progression of refractive error was lower in crocetin group, suggesting a potential protective effect. Although the degree of axial elongation suppression was modest compared to pharmacological or optical treatments, crocetin’s ease of use and favourable safety profile make it a compelling option especially for children during critical phase of eye growth and its suitability of combination therapy or for use in settings where other interventions may be impractical adds to its appeal.

### ABCA4-Related Stargardt Macular Dystrophy (STGD1)

Piccardi et al.^21^ RCT involved 31 patients diagnosed with ABCA4-related STGD1 and a visual acuity above 0.25. Participants received either oral saffron (20mg/day) or a placebo daily for six months, then switched to the opposite treatment for another six months. The study fERG amplitude findings found saffron’s treatment maintained stable retinal responses and visual acuity with no notable changes observed across treatment phases. Despite this saffron appeared to preserve retinal function, indicating a potential neuroprotective effect on photoreceptors and fERG amplitude remained stable after saffron treatment but showed a slight decline after placebo.

### Refractory Diabetic Macular Edema (DME)

Sepahi et al.^22^ study involved 60 patients (101 eyes) with DME that had not responded to conventional treatments such as anti-vascular endothelial growth factor agent (anti-VEGF) therapy, corticosteroids or laser procedures. The treatment groups were provided various dosages, Group 1: crocin 5mg/day; Group 2: crocin 15mg/day and Group 3: placebo for three months.

Key outcomes revealed patients receiving 15mg/day of crocin showed significant improvements in both visual acuity and macular thickness, indicating a dose-dependent benefit. A decrease in HbA1c levels was also observed suggesting possible systemic effects that could contribute to retinal health. While the 5mg crocin dose showed clinical improvements in HbA1c, FBS, CMT and BCVA, these changes were not statistically significant relative to the placebo. Its dual impact on retinal anatomy and systemic glycaemic control treatments, reveals crocin as a promising candidate for integrated diabetes and vision care.

## Discussion

This review examined clinical trials assessing saffron for ocular health and found that saffron was a safe and potentially effective for different ocular diseases and conditions. These results are supported by evidence from various AMD studies, showing saffron supplementation consistently demonstrated functional benefits such as Lashay et al.^23^ who observed enhanced ERG responses in both wet and dry AMD short term. Participants in this study showed enhanced BCVA and improved retinal sensitivity within 3 months suggesting saffron has the potential as a rapid acting neuroprotective agent. Though effects diminished by six months, this study adds to the growing body of evidence supporting saffron as a safe and effective adjunct for managing early-stage AMD. Although Lashay et al.^23^ found that saffron only worked for a short period of time for AMD, other research found that sustained benefits from saffron supplementation were maintained for up to 12 months.^14,17^ In comparison, Broadhead et al (2019) reported significant improvements in visual acuity and reduced multifocal electroretinogram (mfERG) latency following 20 mg/day saffron for three months. These effects were sustained in a 12-month open-label extension,^15^ suggesting long-term retinal support. Riazi et al (2017) found that 50 mg/day saffron over three months significantly improved visual acuity and contrast sensitivity in dry AMD patients.

Heitmar et al.^6^ narrative review, attributed benefits to saffron’s antioxidant and neuroprotective properties in retinal function preservation. The multiple randomized controlled trials showed that saffron supplementation typically 20-50 mg/day improved retinal function, contrast sensitivity and visual acuity in early AMD patients. Benefits were observed within 3 months and sustained with continued use. The main significant improvements were found in visual acuity and reduced mfERG latency. The differences in the length of time the participants experienced benefits from the studies may come down to the raw ingredients used in the products such as whole saffron versus an extract, the quality of the product, and form it came in such as capsules versus powder. Overall, all studies concluded that saffron was found to be beneficial for people with AMD and if the benefits were not sustained, it is safe for them to continue taking the supplement long term.

There are many other mixed supplements on the market that include saffron as an ingredient which have also found benefit such as Macumax, a multi-nutrient which includes the ingredients, Lutein, Zeaxanthin, Bilberry, Zinc and Saffron.^18^ No comparison effective studies have been identified comparing saffron only to a multi-nutrient supplement to see if one is better than the other, however, as both were found to be safe and effective, AMD patients might consider trying different nutraceutical ingredients to determine which ones work best for them.

Another ocular disease that showed promising effects from saffron and crocin is POAG. This review found a study that showed improvement in both IOP and cup to disc ratio, with crocin 15mg/day for six weeks.^3^ Similar results were found with saffron supplementation for lowering IOP as well as being beneficial for ocular hypotensive effects which could potentially support its use as adjunct therapy in glaucoma management.^24^ These studies did not look at sustained benefit, so it’s difficult to say if people with POAG would need to continue taking the supplement for it to have benefit or if there is sustained benefit after around six weeks of supplementation.

One of the interesting findings was the benefit of saffron in diabetes. Saffron has demonstrated dual efficacy in diabetes by improving systemic metabolic parameters and protecting against ocular complications.^22^ Metabolically saffron was found to enhance glucose uptake while reducing oxidative stress via decreasing fasting plasma glucose (FPG), HbA1c and reducing insulin sensitivity. In the ocular domain its mechanisms were found to have neuroprotective and anti-VEGF effects.^22^ Although a meta-analysis study^25^ showed that all major anti-VEGF agents for neovascular AMD provided comparable visual benefits and safety profiles, indicating that any future treatment including nutraceuticals such as saffron must demonstrate improvements beyond this consistent baseline to be clinically meaningful. By comparison, Chen et al.^26^ study relating to Diabetic retinopathy (DR) showed that myopia particularly when accompanied by greater axial length, appears to protect DR while hyperopia increases risk, highlighting refractive status as a meaningful nonglycemic modifier of DR whilst underscoring the importance of lifelong ocular health progression when assessing DR including DME.

Another compelling study^27^ found that metformin protects the retina from diabetes-related injury, by restoring mitochondrial function and activating the glyoxalase detoxification pathway offering a mechanistic guideline to explore saffron’s antioxidant and anti-inflammatory actions within a broader therapeutic approach, targeting mitochondrial dysfunction across retinal conditions. A review conducted in 2024 found that participants received saffron supplements ranging from 5mg/day to 1g/day over a period of 8 to 12 weeks and found that FPG decreased by an average of 8.42mg/dL compared to placebo, and HbA1c levels dropped by 0.22%, indicating improved glycaemic control.^28^ Therefore, saffron could serve as a promising adjunct therapy for diabetes for both ocular and systemic complications.

Looking at Saffron’s dosing, there are a number of variations used from this review. Generally, the main dose range identified was 15mg–50gm daily for approximately 3-6 months. The majority of studies on average used 20mg/day of saffron daily which is comparable to Heitmar et al.^6^ narrative review which found most studies used 15-30 mg/day orally. When examining specific ocular disease conditions variations may occur due to patient ages, different disease mechanisms, therapeutic standards and appear to require different levels of crocin, crocetin, including related bioactive compounds to achieve a measurable effect. AMD was found to benefit from 20mg/day shown to improve retinal function and visual acuity,^14,15,17,23^ POAG 15mg/day the need for neuroprotective and vascular effects without extreme systemic influence,^18^ DME 15g/day is sufficient to modulate retinal edema and metabolic stress,^22^ ABCA4-related STGD1 20mg/day is consistent with higher oxidative and photoreceptor stress^21^ and CSR 15mg/day suitable for modulating choroidal perfusion and lowering oxidative stress.^19^ Lower doses were also used for PM patients, such as crocetin 7.5mg/day reflecting children’s lower body mass compared to adults and different therapeutic treatment of slowing axial elongation rather than restoring retinal tissue,^20^ Liu et al^28^ indicated dosage efficacy appeared dose dependent and optimal dosing depends on patient’s characteristics, underlying pathology and mechanism of action required.

The studies found that saffron and its bioactive compounds, crocin, crocetin and safranal, exhibit a multifaceted protective effect on ocular health and the mechanisms of action for these bioactive compounds are, anti-inflammatory which suppress microglial activation of cytokine release to help maintain retinal health; Neuroprotective actions, preserving retinal ganglion cells and photoreceptors; Vascular support, enhancing retinal blood flow which reduces VEGF-mediated leakage; Functional improvement, by boosting mfERG, contrast sensitivity and acuity; Antioxidants, reducing oxidative stress in retinal cells. In comparison, Sekar et al.^29^ demonstrated that Saffron’s neuroprotective effect is shown to stem from its ability to inhibit P2X7-receptor driven calcium overload and ATP induced cytotoxicity, which are key processes that trigger microglial activation and photoreceptor stress in retinal diseases such as glaucoma and AMD, revealing that saffron directly modulates inflammatory pathway rather than acting as a nonspecific antioxidant. Whilst saffron and its key constituents exhibit antioxidant activity the precise molecular pathways that influence retinal cells are still not understood, although Cerdá-Bernad et al.^30^ suggests that the constituents may modulate oxidative stress responses, stabilse mitochondrial function and influence gene expression related to photoreceptor viability. However, the direct mechanistic interactions with retinal tissues have not been thoroughly elucidated. More studies are required to explain how saffron’s bioactive compounds enter ocular tissue and modulate retinal signalling pathways.

The review has found that saffron supplementation was safe, similarly to Lashay et al.,^23^ Lui et al.^28^ and Bonyadi et al.^24^ who found that saffron was well tolerated with no serious adverse events reported in various ocular conditions. Heitmar et al.^6^ narrative review also found that saffron was well tolerated across all reviewed studies with no serious adverse effects reported and minor side effects included mild gastrointestinal discomfort or headache, which were transient. For glaucoma, Udensi et al.^31^ study did not report any adverse events, reinforcing the safety profile of macular pigment carotenoids in glaucoma patients over an extended period of time.

Saffron and its derivatives are remarkably safe across a wide range of applications from ocular health, diabetes and neuroprotective adjuncts. Their low toxicity, high tolerability and selective bioavailability make them ideal candidates for functional foods, supplements and therapeutic agents. Saffron’s key constituents are consistently reported as safe across clinical and preclinical studies with the compound Saffron well tolerated from 5-50mg/day with mild gastrointestinal disturbance in rare cases. Crocin has safe low systemic toxicity, is water soluble, poor bioavailability but no adverse effects. Crocetin rapidly absorbed lipophilic, light sensitive and requires proper storage. Safranal safe in low doses, may cause mild sedation or hypotension at high doses. Overall, no major adverse reactions reported across ocular, metabolic and neuroprotective trials, high doses >50mg/day may cause dizziness, nausea, mild gastrointestinal discomfort or headache, with no hepatoxicity, nephrotoxicity or retinal damage observed.

### Limitations

This literature review has several limitations that warrant consideration. The scope of the review excluded non-English publications which may have contained relevant findings. Additionally, the lack of heterogeneity reduced the ability to be able to conduct a meta-analysis. Future research would benefit from standardization of outcome measures and research designs.

## Conclusion

*Crocus sativus* (Saffron) and its active constituents crocin and crocetin demonstrate promising therapeutic potential in ophthalmology, offering neuroprotective, anti-inflammatory, and antioxidant benefits across a spectrum of ocular diseases. Their consistent efficacy in improving visual acuity, retinal function, and intraocular pressure combined with favourable safety profiles supports their integration and adjunct therapy strategies. Optimal dosage appears condition specific, dispensed as an oral supplementation, with crocin at 15mg/day, crocetin 7.5mg/day for paediatrics, and saffron at 20-50mg/day, showing the most pronounce effects.

There is a growing need to clarify the appropriate use and ideal dosing of saffron for different ocular conditions and different therapeutic forms for dispensing, as current evidence suggests potential benefits but lacks standardized, condition specific guidelines. Future large scale multicentred trials with standardized endpoints and longer follow-up periods are warranted to confirm these findings, elucidate mechanism of action, refine dosing protocols and safety, tailored to the individual ocular pathologies.
